# Allopathic Suppression of Lochia

**Published:** 1893-04

**Authors:** D. C. McLaren

**Affiliations:** Ottowa, Can.


### ALLOPATHIC SUPPRESSION OF LOCHIA.

D. C. McLaren, M. D., Ottawa, Can.

This case is that of a lady about thirty-five years of age, whose second child was born on August 1st, 1885. Up to this date she had always been a strong, healthy woman, but, after a few days, the foul condition of the lochia excited the combined animosity of physician and nurse, and an intra-uterine injection of Iodine was ordered and faithfully administered. This promptly dried up the discharge, but was immediately followed by a sense of fullness in the abdomen ; this sensation soon became reality, and for some months she was as much distended as if again pregnant. Severe headaches came on in November, for which she was treated by the allopathic methods in vain. Finally, a specialist discovered a lacerated cervix, and operated. The headaches left, but in place of them came a most distressing oppression across the chest ; this was so bad at one time that she was almost out of her mind. This trouble was at its height in March, 1890, when she had a miscarriage. She came to me in October, 1890, weeping bitterly as she described her symptoms, saying she surely had heart disease, that she was possessed by the devil, etc. She complained so much about her heart that it was examined, but found to be sound ; there was, however, a decided throbbing at the pit of the stomach, which was at times visible externally. This spot was exceedingly tender on pressure, and there seemed to be a lump beneath. I hesitate, however, to declare that it was an aneurism, because, in our provings of Iodine, there occurs the symptom : " Violent throbbing of the abdominal aorta." I tried several apparently indicated remedies, but Lycopodium was the first to do any real good ; its principal indication was the formation of flatulence in the stomach and relief from eructation. The course of the treatment was interrupted in March, 1891, by another miscarriage, for which the lady's husband, in his haste, called in the former allopathic physician. During the summer I gave one dose of Iodum high, but cannot record any great improvement from it. I became convinced that pregnancy would do this case much good, and when she was again threatened with miscarriage, in October, I was promptly on hand, and stopped the manifestation with a single dose of Sabina$^{200}$. Subsequent symptoms required, from time to time, a dose of *Belladonna*, but during the last three months of pregnancy she was left under the influence of *Psorinum*. Her baby was born on Good Friday, 1892, and the lochial discharge for a week was most astonishingly profuse. She recovered well, and the pulsation at the stomach pit has entirely disappeared. I do not claim to have cured a case of aneurism, but to have in some degree overcome the results of the original allopathic suppression.

DISCUSSION.

Dr. Farley—The trouble in that case suggests to me a case I had last week. A physician in my town asked me to see a case for him, which he called asthma. He had given several remedies. She had long been a sufferer from hemorrhoids, and, I judge, also a fissure. She had been operated on for rectal trouble, and soon after had the asthma. I gave Kali-bichrom.$^{200}$, in water, at intervals of two hours for twenty-four hours. The respiration was better, and she was sitting erect on the next day. It was a marked improvement, but her rectum was getting sore, and in a short time her asthma was gone, but her rectum was again the seat of much suffering. The character of the expectoration drew my attention to the remedy.

---

The following amendment to the By-Laws was presented by Dr. Bell to be acted upon at the next meeting :

"The order of business may be changed at any annual meeting by a unanimous vote."

The following amendment to the By-Laws was presented by Dr. Custis to be acted upon at the next meeting :

"The Association shall meet annually at such time and place as may be fixed upon by ballot at the annual meeting. If suitable arrangements cannot be made in accordance with the instructions given, the Executive Committee may, with the approval of a majority of the members, change the place of meeting. The ballot to be taken by mail, the members to have the choice of two places named by the committee."

The motion was made and carried that the Executive Committee have charge of the publication of the Transactions.

A vote of thanks was passed to the proprietor of the hotel.

The minutes of the meeting were read by the Secretary and were approved.

Adjourned *sine die* at 11.30 A. M.

